# What Is the Appropriate Antibiotic Administration During Tooth Extractions in Patients Receiving High-Dose Denosumab?

**DOI:** 10.7759/cureus.67237

**Published:** 2024-08-19

**Authors:** Eiji Iwata, Takumi Hasegawa, Hiroaki Ohori, Toshiya Oko, Tsutomu Minamikawa, Daisuke Miyai, Masaki Kobayashi, Naoki Takata, Shungo Furudoi, Junichiro Takeuchi, Kosuke Matsumoto, Akira Tachibana, Masaya Akashi

**Affiliations:** 1 Oral and Maxillofacial Surgery, Kakogawa Central City Hospital, Kakogawa, JPN; 2 Oral and Maxillofacial Surgery, Kobe University Hospital, Kobe, JPN; 3 Oral and Maxillofacial Surgery, Saiseikai Hygoken Hospital, Kobe, JPN; 4 Oral and Maxillofacial Surgery, Kita-Harima Medical Center, Ono, JPN; 5 Oral and Maxillofacial Surgery, Nishiwaki Municipal Hospital, Nishiwaki, JPN; 6 Oral and Maxillofacial Surgery, Shin-suma General Hospital, Kobe, JPN; 7 Oral and Maxillofacial Surgery, Hyogo Prefectural Awaji Medical Center, Sumoto, JPN; 8 Oral and Maxillofacial Surgery, Konan Medical Center, Kobe, JPN; 9 Oral and Maxillofacial Surgery, Hyogo Prefectural Tamba Medical Center, Tamba, JPN; 10 Oral and Maxillofacial Surgery, Kobe Central Hospital, Kobe, JPN

**Keywords:** current-status, perioperative antibiotic administration, tooth extraction, denosumab, medication-related osteonecrosis of the jaw

## Abstract

Purpose: Medication-related osteonecrosis of the jaw (MRONJ) occasionally occurs following tooth extractions in cancer patients receiving denosumab (Dmab). However, there are currently no established guidelines for perioperative antibiotic administration during tooth extraction in these patients. The primary objective was to develop guidelines for the dose and frequency of antibiotics during tooth extraction by investigating the correlation between the current status of antibiotic administration and the development of MRONJ.

Methods: This study included 68 cancer patients receiving high-dose Dmab who had tooth extractions between 2012 and 2022 at 10 hospitals. The relationship between the way of perioperative antibiotic administration and the development of MRONJ was analyzed. A *P-*value < .05 was considered significant.

Results: There was considerable variability across hospitals and surgeons regarding the type, dosage, and duration of antibiotic administration. Amoxicillin (AMPC) was the most commonly used antibiotic. Focusing exclusively on teeth extracted under AMPC administration, MRONJ developed in 21 out of 123 teeth (17.0%). No significant relationship was found between the development of MRONJ and the dosage or duration of perioperative AMPC administration.

Conclusion: Perioperative antibiotic administration alone may not be sufficient to prevent MRONJ. Therefore, a single preoperative dose is likely adequate for effective and appropriate AMPC administration.

## Introduction

Denosumab (Dmab) is a human monoclonal antibody that targets the receptor activator of nuclear factor-κB ligand (RANKL) and represents a breakthrough in the treatment of osteoporosis, multiple myeloma, bone metastases of solid cancers, and giant cell tumors of bones [1,2]. Dmab targets RANKL and inhibits the binding of RANKL to RANK. It inhibits osteoclast maturation, activation, and function by binding to RANKL, with the result being a reduced rate of bone resorption [3,4]. Compared to bisphosphonates (BPs), which directly inhibit osteoclast activity, Dmab offers a safer alternative for patients with renal impairment. It has a much shorter half-life than BPs because it is not embedded in bone tissue, and it exhibits a more delayed onset of skeletal-related events [5-7]. In a double-blind phase III trial, overall survival, disease progression, and the rate of adverse events were similar between groups receiving Dmab and those receiving BPs [8]. Consequently, the use of Dmab has recently increased.

Medication-related osteonecrosis of the jaw (MRONJ) is a well-known serious side effect of anti-resorptive agents (ARAs) such as BPs and Dmab [9]. Studies have shown that the risk of developing MRONJ in patients receiving high-dose ARAs is higher than in those receiving low-dose ARAs [10-13]. Additionally, reports indicate that the risk of developing MRONJ in patients treated with high-dose Dmab is greater than in those treated with zoledronate [14-16]. In more detail, Ikesue et al. reported that the development of MRONJ was significantly higher in the high-dose Dmab group than in the zoledronate group (9.6% vs. 4.8%, *P* = 0.009) [14]. Jiang et al. also reported similar results (risk ratio 1.41; 95% confidence interval 1.01-1.95; *P* = 0.04) by meta-analysis [16]. These findings suggest that high-dose Dmab carries the highest risk of MRONJ among ARAs. 

Tooth extraction is widely recognized as a risk factor for developing MRONJ [9]. Typically, antibiotics are administered prophylactically to prevent postoperative infections following surgeries, including tooth extractions. However, even the recent position paper from the 2022 American Association of Oral and Maxillofacial Surgeons (AAOMS 2022), which is widely referenced, lacks specific guidelines for antibiotic use during tooth extractions [17]. Similarly, the latest position paper by the 2023 Japanese Allied Committee on Osteonecrosis of the Jaw (JACOJ 2023) [18] does not establish clear criteria but recommends prudent antibiotic usage akin to that for invasive dental procedures, referencing the Japanese Clinical Practice Guidelines for Antimicrobial Prophylaxis in Surgery (JCOPG-APS 2016) [19]. The JCOPG-APS 2016 guidelines provide recommendations for the types of antibiotics and the duration of administration for various surgeries. The JCOPG-APS 2016 guidelines categorize patients undergoing tooth extractions into four groups: extraction of mandibular impacted tooth; tooth extraction in patients with high risk factors of infective endocarditis (IE); tooth extraction in patients with risk factors of surgical site infections (SSIs); tooth extraction in patients without risk factors of IE or SSIs. The type, dosage, and duration of antibiotic administration are different for each group and it remains unclear which group tooth extraction in patients receiving ARAs applies to. Therefore, this study aims to develop criteria for perioperative antibiotic administration during tooth extractions by examining the relationship between the current status of antibiotic administration and the development of MRONJ in patients receiving high-dose Dmab across multiple centers.

## Materials and methods

Patients

This study included a total of 68 cancer patients who received high-dose Dmab and underwent tooth extractions at 10 hospitals (Kakogawa Central City Hospital, Kakogawa; Kobe University Hospital, Kobe; Saiseikai Hygo-ken Hospital, Kobe; Kita-Harima Medical Center, Ono; Nishiwaki Municipal Hospital, Nishiwaki; Shin-suma General Hospital, Kobe; Hyogo Prefectural Awaji Medical Center, Sumoto; Konan Medical Center, Kobe; Hyogo Prefectural Tamba Medical Center, Tamba; Kobe Central Hospital, Kobe) in Japan from January 2012 to December 2022. The inclusion criteria were as follows: patients aged 18 years and older and those receiving 120mg of Dmab every four weeks to treat bone metastases of solid cancers or multiple myeloma. The exclusion criteria included patients who already had MRONJ (e.g., extraction of teeth with surrounding exposed bone) at the time of tooth extraction, those with a history of radiation therapy to the jaws or metastatic disease to the jaws, and patients who declined to participate after the publication of this study.

Data collection

Data were retrospectively collected from medical records, covering variables such as patient sex, age, type of cancer, chemotherapy, diabetes, steroid use, duration of Dmab administration, drug holiday before extraction, MRONJ staging at diagnosis, and the specifics of perioperative antibiotic administration. The definition and staging of MRONJ adhered to the AAOMS 2022 staging system [17]: briefly, exposed bone or bone that can be probed through an intraoral or extraoral fistula in the maxillofacial region and has persisted for longer than eight weeks; stage 1 was characterized by exposed bone without any symptoms; stage 2 by exposed bone with infection; and stage 3 by exposed bone with complications such as pathological fracture, extraoral fistula, or osteolysis extending to the inferior border of the mandible or the sinus floor. Regarding stage 0, there are differing opinions; it was excluded in the JACOJ 2023 [18] and also in this study.

Ethics approval and consent to participate

This study was conducted in accordance with the 1964 Declaration of Helsinki. Ethical approval was obtained from the Institutional Review Board (IRB) of Kakogawa Central City Hospital (authorization number: 2022-20). The ethics committee approved the study and gave us administrative permission to access the data used in this study. Since this was a retrospective study, the research plan was published on the homepage of the participating hospitals according to the instructions of the IRB, in accordance with the guaranteed opt-out opportunity.

Statistical analysis

All statistical analyses were conducted using Ekuseru-Toukei 2016 (Social Survey Research Information Co., Ltd.; Tokyo, Japan). The relationship between each variable and the development of MRONJ was examined using Fisher's exact test or the chi-squared test for categorical variables. Statistical significance was established at *P* < 0.05.

## Results

Investigation of all teeth

**Figure 1:**
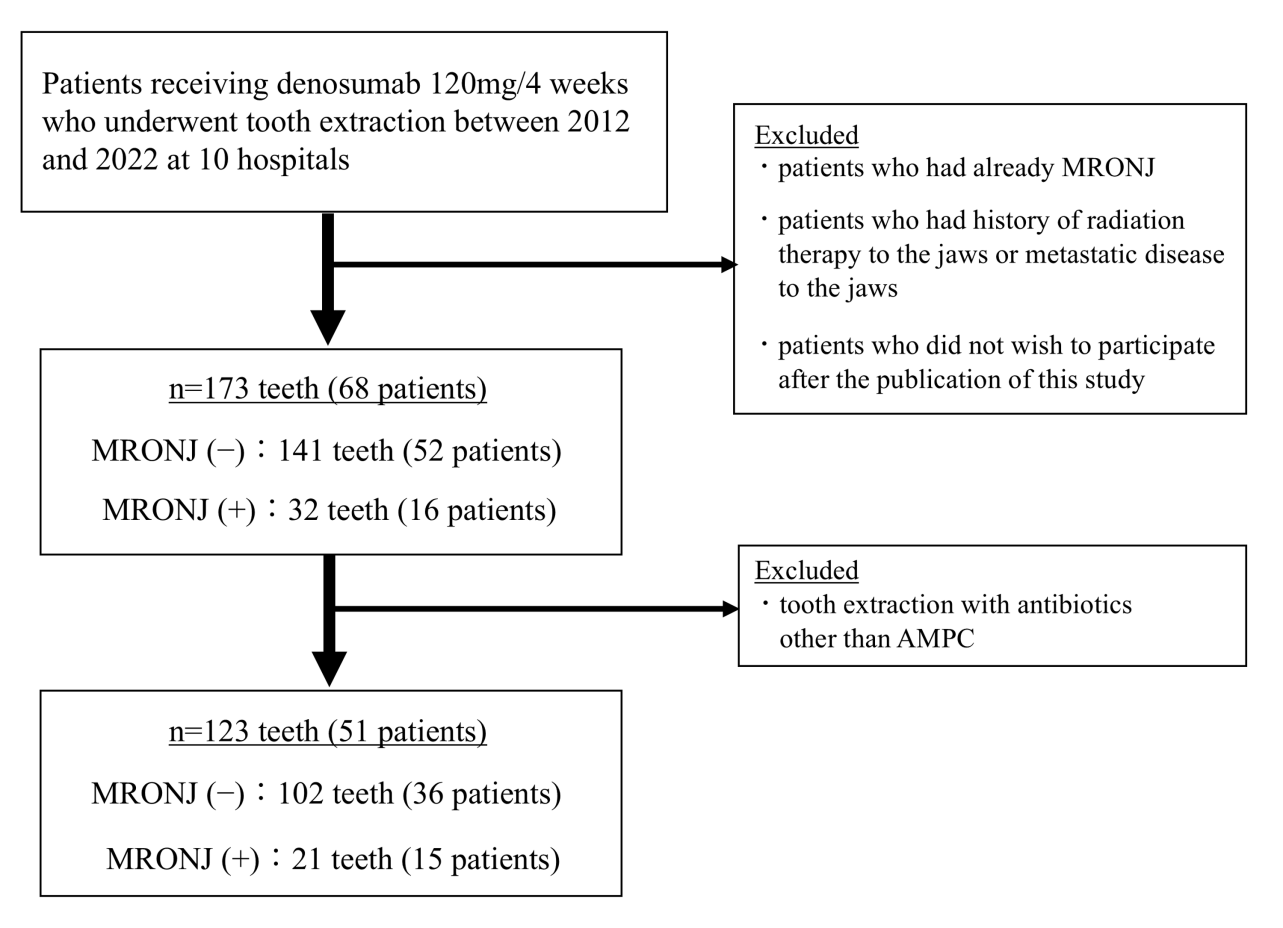
Subject registration chart and number of teeth AMPC: Amoxicillin; MRONJ: medication-related osteonecrosis of the jaw

**Figure 2:**
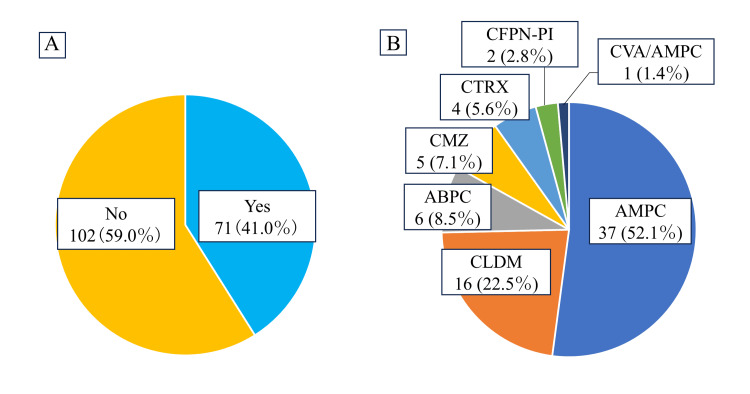
Content of preoperative antibiotic administration (A) Percentage of teeth extracted with preoperative antibiotic administration. (B) Species of antibiotics in procedures which were administered antibiotics (total 71 procedures). AMPC: Amoxicillin; CLDM: clindamycin; ABPC: ampicillin; CMZ: cefmetazole; CTRX: ceftriaxone; CFPN-PI: cefcapene pivoxil; CVA/AMPC: clavulanic acid/ amoxicillin

MRONJ developed after the extraction of 32 out of 173 teeth (18.4%) in 68 patients (Figure 1). Figure 2 illustrates the content of preoperative antibiotics. Teeth extracted with preoperative antibiotics were less common than those without (41.0% vs 59.0%). There was a lack of uniformity in the type and dosage of antibiotic administration across hospitals and surgeons; however, the most commonly used antibiotic was amoxicillin (AMPC) at 52.1%, followed by clindamycin (CLDM) at 22.5%. Intravenous antibiotics were administered during extraction of 15 out of 71 teeth (21.2%). The dose of antibiotic in each group was as follows: AMPC: 250 mg or 500 mg or 1000 mg per time; CLDM: 600 mg per time; ampicillin (ABPC): 1000 mg per time; cefmetazole (CMZ): 1000 mg per time; ceftriaxone (CTRX): 1000 mg per time, cefcapene pivoxil (CFPN-PI): 100 mg per time; clavulanic acid/amoxicillin (CVA/AMPC): 250 mg per time (data not shown).

**Figure 3:**
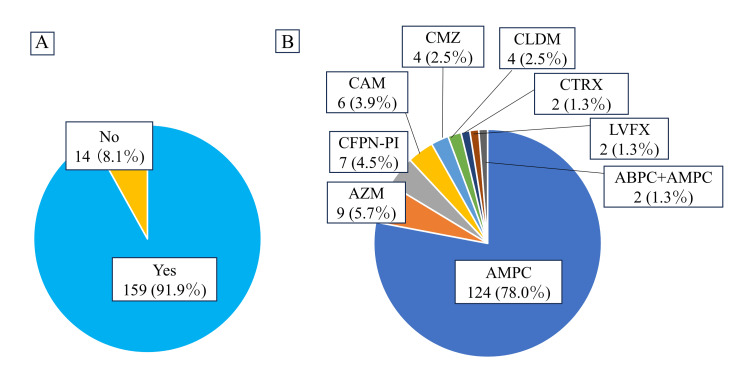
Content of postoperative antibiotic administration (A) Percentage of teeth extracted with postoperative antibiotic administration. (B) Species of antibiotics in procedures which were administered antibiotics (total 159 procedures). AMPC: Amoxicillin; AZM: azithromycin; CFPN-PI: cefcapene pivoxil; CAM: clarithromycin; CMZ: cefmetazole; CLDM: clindamycin; CTRX: ceftriaxone; LVFX: levofloxacin; ABPC+AMPC: ampicillin+amoxicillin

Figure 3 details postoperative antibiotic administration, showing that a significantly higher number of teeth extracted with postoperative antibiotic administration (91.9% vs 8.1%). There was a lack of uniformity in the type, dosage, and duration of antibiotic administration across hospitals and surgeons; however, AMPC was by far the most commonly used antibiotic (78.0%). The dose of antibiotic in each group was as follows: AMPC: 500 mg or 750 mg or 1000 mg per day; azithromycin (AZM): 500 mg per day; clarithromycin (CAM): 400 mg per day; CMZ: 2000 mg per day; CLDM: 1200 mg per day; CTRX: 2000 mg per day, levofloxacin (LVFX): 500 mg per day; ABPC+AMPC: 1000 mg+750 mg per day (data not shown).

Table 1 examines the relationship between perioperative antibiotic administration and the development of MRONJ. There was no significant difference in the dosage of "both pre-operative and postoperative antibiotics administration" and the development of MRONJ, nor was there a difference in the dosage of "only postoperative antibiotics administration". In this study, we could not analyze the effect of preoperative antibiotics administration because there are almost no patients with only those. Regarding the duration of antibiotic administration, no significant difference was found in the development of MRONJ, regardless of whether preoperative antibiotics were administered. Of the 71 teeth where antibiotics were administered for longer than three days, MRONJ developed in 11 teeth (15.5%). The longest duration of antibiotic administration was seven days, and in these instances, MRONJ did not develop.

**Table 1:** The relationship between perioperative antibiotic administration and development of MRONJ ^a^Fisher’s exact test; ^b^Chi-squared test; MRONJ: medication-related osteonecrosis of the jaw

Variables		MRONJ (+) (n=32)	MRONJ (-) (n=141)	*P-*value	Between-group comparison
Both pre- and postoperative antibiotic administration	Yes (total)	19 (59.4%)	43 (30.5%)		
	Oral → Oral	12 (63.1%)	22 (15.6%)	0.336^ a^	Among four groups
	Intravenous → Oral	3 (9.5%)	14 (9.9%)		
	Intravenous → Intravenous	4 (12.5%)	5 (3.6%)		
	Intravenous → Intravenous and oral	0 (0.0%)	2 (1.4%)		
Only postoperative antibiotics administration	Yes (Total)	10 (31.3%)	87 (61.7%)		
	Intravenous	0 (0.0%)	0 (0.0%)	―	Intravenous vs Oral
	Oral	10 (31.3%)	87 (61.7%)		
Duration of antibiotics administration	Both pre- and postoperative (total)	19 (59.4%)	43 (30.5%)		
	2 days	13 (40.6%)	28 (19.9%)	0.633^ b^	Among three groups
	3 days	6 (18.8%)	13 (9.2%)		
	7 days	0 (0.0%)	2 (1.4%)		
	Only postoperative (total)	10 (31.3%)	87 (61.7%)		
	1–2 days	5 (15.7%)	42 (29.8%)	0.164^ b^	Among three groups
	3 days	4 (12.5%)	44 (31.2%)		
	4–5 days	1 (3.1%)	1 (0.7%)		

Investigation of only teeth extracted under AMPC administration

Focusing exclusively on teeth extracted under AMPC administration, MRONJ developed in 21 out of 123 teeth (17.0%) in 51 patients (Figure 1). The clinical characteristics of the patients are presented in Table 2. The median age was 69.0 years, with breast cancer being the most common type of cancer, followed by lung cancer. The median duration of Dmab administration was 6.0 months, with a range from 0 to 108 months. The majority of patients underwent tooth extractions without a drug holiday prior to the extraction (92.2%). Most of the MRONJ cases were diagnosed at stage 1.

**Table 2:** Clinical characteristics of patients MRONJ: Medication-related osteonecrosis of the jaw

Variables		N=51
Sex	Male	25 (49.0%)
	Female	36 (51.0%)
Age (years)	Median (range)	69.0 (23-88)
Type of cancer	Breast cancer	18 (35.3%)
	Lung cancer	12 (23.5%)
	Prostate cancer	9 (17.6%)
	Thyroid cancer	2 (3.9%)
	Multiple myeloma	2 (3.9%)
	Others	8 (15.7%)
Chemotherapy	Yes	23 (45.1%)
	No	28 (54.9%)
Diabetes	Yes	9 (17.6%)
	No	42 (81.4%)
Steroid use	Yes	9 (17.6%)
	No	42 (81.4%)
Duration of denosumab administration (months)	Median (range)	6.0 (0-108)
Drug holiday before extraction	Yes	4 (7.8%)
	No	47 (92.2%)
Patients with MRONJ	Total	15 (29.4%)
MRONJ Stage when diagnosed	Stage 1	9 (17.6%)
	Stage 2	6 (11.8%)
	Stage 3	0 (0.0%)

Table 3 details the relationship between perioperative AMPC administration and the development of MRONJ. No significant difference was observed between the dosage of "both pre- and postoperative AMPC administration" and the development of MRONJ. Similarly, no significant difference was found in the daily dosage of "only postoperative AMPC administration". Unfortunately, we could not analyze the effect of preoperative AMPC administration because there are almost no patients of only those. Regarding the duration of AMPC administration, there was also no significant correlation with the development of MRONJ. Of the teeth extracted AMPC administration for more than three days, MRONJ developed in 4 of 47 teeth (8.5%).

**Table 3:** The relationship between perioperative AMPC administration and development of MRONJ administration ^a^Chi-squared test; ^b^Fisher’s exact test; AMPC: amoxicillin; MRONJ: medication-related osteonecrosis of the jaw

Variables		MRONJ (+) (n=21)	MRONJ (-) (n=102)	*P-*value	Between-group comparison
Both pre- and postoperative AMPC administration	Yes (total)	11 (52.4%)	23 (22.5%)		
	250mg/time → 750mg/day	2 (9.5%)	9 (8.8%)	0.269^ a^	Among four groups
	250mg/time → 1000mg/day	3 (14.3%)	4 (3.9%)		
	500mg/time → 750mg/day	6 (28.6%)	7 (6.9%)		
	1000mg/time → 750mg/day	0 (0.0%)	3 (2.9%)		
Only postoperative AMPC administration	Yes (total)	7 (33.3%)	74 (72.5%)		
	500mg/day	0 (0.0%)	9 (8.8%)	1.000^ b^	500 mg/day vs 750 mg/day
	750mg/day	7 (33.3%)	65 (63.7%)		
Duration of AMPC administration	Both pre- and postoperative (total)	11 (52.4%)	23 (22.5%)		
	2 days	9 (42.9%)	19 (18.6%)	1.000^ b^	2 days vs 3 days
	3 days	2 (9.5%)	4 (3.9%)		
	Only postoperative (total)	7 (33.3%)	74 (72.5%)		
	1–2 days	5 (23.8%)	35 (34.3%)	0.264^ b^	1–2 days vs 3–4 days
	3–4 days	2 (9.5%)	39 (38.2%)		

## Discussion

Tooth extraction is widely recognized as a common risk factor for developing MRONJ [9,17]. However, there are currently no established guidelines for perioperative antibiotic administration in patients undergoing tooth extractions who are receiving ARAs. This study aimed to develop such criteria by examining the existing practices of antibiotic administration during tooth extractions in patients receiving high-dose Dmab and their correlation with the development of MRONJ. The findings revealed a lack of uniformity in the type, dosage, and duration of antibiotics administered across different hospitals and surgeons. There was no significant association between the development of MRONJ and either the dosage or the duration of perioperative antibiotic administration in 173 teeth. AMPC was the most frequently used antibiotic, accounting for 78.0%. Even when focusing solely on the teeth extracted under AMPC administration, there was no significant association between the development of MRONJ and both the dosage and duration of perioperative AMPC administration in 123 teeth.

There are up to 1000 species of organisms capable of colonizing the oral cavity [20]. The oral environment harbors various bacteria that can potentially cause infections in wounds after tooth extractions [21]. When tooth extraction, the area becomes more vulnerable to infection even with proper cleaning and oral hygiene. In addition, if the tooth extracted had been already infected, bacteria may have spread to the surrounding tissues, increasing the risk of infection. Antibiotics are effective in treating such infections and can prevent the development of wound infections. Common antibiotics used to treat or prevent oral infections include AMPC, erythromycin, CLDM, and metronidazole [21]. These antibiotics function by killing or slowing the growth of bacteria responsible for infections. However, some infections may resolve on their own. Moreover, unnecessary administration of antibiotics can lead to reduced effectiveness in the future [21], contributing to the global issue of antimicrobial resistance. The most recent position paper on MRONJ, the JACOJ 2023, recommends appropriate antibiotic use similar to that for invasive dental treatments, referencing the JCOPG-APS 2016 [19]. The JCOPG-APS 2016 guidelines suggest prophylactic antibiotic administration for patients undergoing the extraction of mandibular impacted teeth, those with high-risk factors for IE such as prosthetic valve replacement or a history of IE, and those with risk factors for SSI such as a body mass index ≥ 25, steroid use, immunosuppressant use, and uncontrolled diabetes (Table 4). Conversely, prophylactic antibiotic administration is not recommended for tooth extractions in patients without risk factors for IE or SSIs. AMPC is recommended as the first-line antibiotic, with CLDM, AZM, or CAM as alternatives for patients allergic to β-lactam antibiotics (Table 4). These are all oral antibiotics. The recommended administration period ranges from a single preoperative dose to a maximum of 48 hours (Table 4). In this study, AMPC was the most commonly used antibiotic, although various antibiotics, including intravenous options, were utilized. The number of teeth extracted with preoperative antibiotic administration was fewer than those without. Approximately 40% of teeth received antibiotics for more than three days; notably, the longest duration was seven days in two teeth. The use of intravenous antibiotics (i.e., high-dose) or extended antibiotic administration may have been employed by surgeons to prevent the development of MRONJ as effectively as possible.

**Table 4:** The part of tooth extraction in the JCOPG-APS 2016 IE: infective endocarditis; SSI: surgical site infection; AMPC: amoxicillin; CVA/AMPC: clavulanic acid/ amoxicillin; ABPC: ampicillin; CLDM: clindamycin; AZM: azithromycin; CAM: clarithromycin.

Surgery species	Recommended antibiotics	Alternative drugs for patients allergic to β-lactam antibiotics	Duration of administration
Extraction of mandibular impacted tooth [19]	AMPC (250mg–1000mg/time) or CVA/AMPC (375mg–1.5g/time)	CLDM	Preoperative single–48 h
Tooth extraction in patients with high risk factors of IE [19]	AMPC (2g/time) or Intravenous ABPC (2g/time)	CLDM, AZM, or CAM	Preoperative single
Tooth extraction in patients with risk factors of SSI [19]	AMPC (250mg–1000mg/time) or CVA/AMPC (375mg–1.5g/time)	CLDM	Preoperative single –48 h
Tooth extraction in patients without risk factors of IE or SSI [19]	No recommended	―	―

The recent systematic review revealed a lack of evidence supporting the efficacy of antibiotics in reducing the risk of developing MRONJ after tooth extractions [22]. The review included 17 studies, 16 of which involved tooth extractions in patients receiving either high-dose or low-dose BPs, and one study focused on patients receiving high-dose Dmab, similar to this study. As for the type of antibiotic used, 2-3 grams of amoxicillin AMPC was the first-line drug in 76.4% of the studies, with 300-600 mg of CLDM as the alternative [22]. In patients receiving BPs, the most common perioperative antibiotic regimen was 2-3 g of AMPC daily, either alone or combined with clavulanate potassium, for 6-7 days [22]. Only one retrospective study examined an appropriate method for tooth extraction in patients receiving high-dose Dmab [23]. This study involved the extraction of 40 teeth from 19 German patients, using a perioperative antibiotic regimen of 10,000,000 IU of intravenous penicillin before surgery and one after teeth extraction. For patients allergic to penicillin, 600 mg of intravenous CLDM was administered three times daily, one day before and one day after the extraction [23]. MRONJ developed in 3 out of 40 teeth (7.5%) [23]. The study suggested this regimen as a criterion for perioperative antibiotic administration [23]. However, the aforementioned systematic review concluded that the data from just one small study was insufficient to determine the regimen’s effectiveness [22]. Many dental conditions that necessitate tooth extraction, such as severe periodontal disease and periapical lesions, often involve bacterial infections in the jawbone. Several reports have suggested that tooth extraction is not the sole cause of MRONJ [24,25]. The Japanese Allied Committee on Osteonecrosis of the Jaw has noted that in some cases, MRONJ may already be latently present before the extraction and becomes manifest afterward [18]. These findings suggest that relying solely on perioperative antibiotic administration is inadequate for preventing the development of MRONJ. Therefore, a single preoperative dose is likely sufficient, considering the need for appropriate antibiotic use.

To the best of our knowledge, this is the first study to investigate the current status of antibiotic administration during tooth extractions in patients receiving high-dose Dmab and its association with the development of MRONJ. However, this study has several limitations. First, it cannot be denied that factors other than antibiotic administration might have influenced the development of MRONJ. A recent study showed that the duration of ARA administration and the presence of a tooth with clinical symptoms were risk factors for development of MRONJ [26]. However, this research intentionally focused on the relationship between the dosage or duration of perioperative antibiotic administration and the development of MRONJ, as our goal was to develop relevant criteria. Further investigations are needed using a prospective study design. Second, the focus of this study and the JCOPG-APS 2016 guidelines is on Japanese patients; therefore, the recommended dosages of antibiotics may differ across different ethnicities [27]. Moreover, no existing position papers on MRONJ provide criteria for perioperative antibiotic administration. The JACOJ 2023 is the only position paper that recommends guidelines for antibiotic administration by referencing another guideline (i.e., the JCPG-APS 2016).

## Conclusions

Our results showed that no significant relationship was found between development of MRONJ and the dosage or duration of perioperative antibiotic. In view of our result and the fact of a lack of evidence supporting the efficacy of antibiotics in reducing the risk of developing MRONJ after tooth extractions, solely relying on perioperative antibiotic administration may be insufficient to prevent the development of MRONJ. Therefore, a single preoperative dose is likely adequate for effective and appropriate AMPC administration.　
